# Pregnancy outcomes after fresh and frozen embryo transfer in women of advanced age and analysis of influencing factors: single-center experience

**DOI:** 10.3389/fendo.2026.1883081

**Published:** 2026-08-12

**Authors:** Hongyan Jia, Jiahui Zhao, Xueqin Li, Ning Yang, Huaiyun Tang, Lisha Tang

**Affiliations:** Lianyungang Maternal and Child Health Hospital, Kangda College of Nanjing Medical University, Lianyungang, China

**Keywords:** advanced age, assisted reproductive technology, live birth, miscarriage, pregnancy

## Abstract

**Objective:**

To investigate the differences in pregnancy outcomes between fresh embryo transfer (ET) and frozen embryo transfer (FET) in women of advanced age undergoing assisted reproductive technology (ART), and to provide evidence for optimizing embryo transfer strategies.

**Methods:**

This retrospective study included 359 women of advanced age (35–45 years) who underwent fresh ET and 605 who underwent FET at the Department of Reproductive Medicine, Lianyungang Maternal and Child Health Hospital from January 2019 to April 2025. Following 1:1 propensity score matching, 288 fresh ET and 288 FET cycles were analyzed. Clinical pregnancy rate, miscarriage rate, live birth rate, preterm birth rate, and neonatal birth weight were compared between the two groups. Multivariable logistic regression was used to identify factors associated with live birth, and conditional logistic regression was performed.

**Results:**

In the matched cohort, miscarriage rate and preterm birth rate were comparable between two groups (both *P* > 0.05). Fresh ET showed a tendency to increase the clinical pregnancy rate (*P* = 0.056) and significantly higher live birth rate (28.13% vs. 20.49%, *P* = 0.033). However, multivariate analysis showed that fresh ET had a higher live birth rate trend than FET (OR = 1.46, *P* = 0.072). Independent factors associated with increased live birth rate were younger female age, transfer of two embryos and transfer good-quality embryos (OR = 0.80, *P* < 0.001; OR = 2.35, *P* < 0.001; OR = 2.25, *P* < 0.017; respectively). These findings were further confirmed in a sensitivity analysis using conditional logistic regression accounting for matched pairs, in which transfer strategy remained non-significant (*P* = 0.344), while female age and transfer of two embryos remained significant predictors. Regarding perinatal outcomes, no significant differences were observed between the two groups for singleton deliveries in terms of gestational age, birth weight, macrosomia, or newborn sex (all *P* > 0.05).

**Conclusion:**

In women of advanced age, the transfer strategy was not an independent predictor of live birth. Instead, younger maternal age and transfer of two embryos were the key factors to improved live birth outcomes.

## Introduction

With the general postponement in childbearing age, the number of advanced-age women undergoing assisted reproductive technology (ART) has been increasing year by year. Advanced age is associated with diminished ovarian reserve, reduced oocyte quantity and quality, and consequently a direct negative impact on ART outcomes (1). The higher rate of embryonic aneuploidy is associated with an increased risk of miscarriage and lower live birth rates in women of advanced age. However, even when transferring euploid embryos, clinical pregnancy and live birth rates remain low in women of advanced age (2), suggesting that factors beyond chromosomal abnormalities play important roles.

During ART treatment, fresh embryo transfer (ET) and frozen embryo transfer (FET) may differentially affect pregnancy outcomes. Fresh ET avoids potential cryoinjury but involves supraphysiological hormone levels that may impair endometrial receptivity (3). In contrast, FET allows more adequate endometrial preparation and reduces the risk of ovarian hyperstimulation syndrome. However, embryos undergo freeze–thaw procedures, and FET may increase the risk of hypertensive disorders and macrosomia (4).

Current evidence on the optimal transfer strategy in low-prognosis populations, including women of advanced age, remains controversial. Some studies suggest fresh ET improves live birth rates (5), while others report no significant difference in live birth rates between fresh ET and FET (6). This study aimed to compare clinical outcomes between fresh ET and FET in women of advanced-age and to analyze factors influencing pregnancy outcomes for each strategy.

## Materials and methods

### Study design and participants

This retrospective study included 359 women of advanced age (aged 35–45 years) who underwent fresh ET and 605 who underwent FET at the Department of Reproductive Medicine, Lianyungang Maternal and Child Health Hospital between January 2019 and April 2025. Clinical indications for ART included advanced age (≥ 35 years), diminished ovarian reserve (DOR, AMH < 0.5 – 1.1 ng/ml or AFC < 5–7 follicles) (7), fallopian tubal obstruction, and ovulation disorders.

Exclusion criteria for fresh ET and FET groups: (1) Patients who failed to obtain oocytes, those with failed fertilization, abnormal fertilization, no transferable embryos, as well as those who did not undergo their first cycle of embryo transfer. (2) Male partner diagnosed with oligoasthenozoospermia; female partner had uterine fibroids, adenomyosis, endometriosis (stage II or higher), moderate-to-severe intrauterine adhesions, or other conditions that affect the uterine cavity. (3) At least one partner had a chromosomal karyotype abnormality. (4) Patients underwent preimplantation genetic testing. (5) Donor sperm or donor oocyte cycles. At our institution, preimplantation genetic testing and oocyte donation programs were not currently available.

### Ovarian stimulation and embryo transfer

Controlled ovarian stimulation protocols included the antagonist protocol, mild stimulation protocol, short-acting GnRH agonist long protocol, and short protocol. The gonadotropin doses were based on indicators such as the women’s age, body mass index (BMI), and ovarian reserve.

Oocyte retrieval was performed under ultrasound guidance. Fertilization was achieved by *in vitro* fertilization (IVF) or intracytoplasmic single sperm injection (ICSI), or rescue ICSI for unfertilized mature oocytes. Cleavage-stage embryo morphology was assessed by evaluating blastomere number and symmetry, degree of fragmentation, presence of multinucleation and vacuoles, as well as zona pellucida morphology (8). Blastocyst morphology was assessed according to the Gardner and Schoolcraft criteria, which evaluate three components: developmental stage, inner cell mass, and trophectoderm quality (8). In our study, the decision between fresh ET and FET was made based on individual patient conditions. Fresh ET was not chosen when women had undergone clomiphene citrate for more than 5 days in mild stimulation cycles, ovarian hyperstimulation syndrome risk, progesterone >1.5 ng/ml on the trigger day, endometrial thickness less than 7mm or irregular endometrial echo pattern. Endometrial preparation protocols for FET included the natural cycle, ovarian stimulation cycle, hormone replacement therapy cycle, and down-regulated hormone replacement therapy cycle. Embryos were transferred on day 3 (cleavage-stage) or day 5 (blastocyst) after endometrial transformation.

### Luteal phase support

In fresh ET cycles, luteal support consisted of oral dydrogesterone tablets (Duphaston, Abbott Biologicals B.V., Netherlands, 20 mg/d) and progesterone soft capsules (Utrogestan, Cyndea Pharma, S.L., Spain, 400 mg/d for antagonist protocol; 200 mg/d for other protocols). In FET cycles, the natural cycle used oral dydrogesterone tablets 20 mg/d; ovarian stimulation cycle used oral dydrogesterone tablets 20 mg/d combined with progesterone soft capsules 200 mg/d; hormone replacement therapy cycle and down-regulated hormone replacement therapy cycle used oral dydrogesterone tablets 20 mg/d combined with progesterone soft capsules 400 mg/d.

### Outcomes

Clinical pregnancy included both intrauterine pregnancy and ectopic pregnancy. Miscarriage was defined as loss of the pregnancy before 28 weeks. Preterm birth referred to delivery at less than 37 weeks of gestation. Live birth was defined as delivering a live infant. Clinical pregnancy rate = number of cycles with clinical pregnancy/number of transfer cycles × 100%. Miscarriage rate = number of cycles with miscarriage/(number of clinical pregnancies − number of ectopic pregnancies) × 100%. Preterm birth rate = number of preterm births/number of live-birth deliveries × 100%. Live birth rate = number of cycles with live birth/number of transfer cycles × 100%. Neonatal birth weight was recorded. Macrosomia was defined as birth weight ≥ 4000 g, and low birth weight (LBW) as < 2500 g (9).

### Statistical analysis

Statistical analysis was performed using SPSS 32.0. Normally distributed continuous data were expressed as mean ± SD (Standard Deviation) and compared by independent samples *t*-test. Non-normally distributed continuous data were presented as median (interquartile range) [M (Q1, Q2)], and compared by Mann-Whitney U test. Categorical variables were expressed as percentage (counts) and compared using the chi-square test or Fisher’s exact test as appropriate. To minimize selection bias, propensity score matching (PSM) was applied with 1:1 nearest-neighbor matching and a caliper of 0.03. Covariate balance was evaluated using absolute standardized mean differences (SMDs), with a value below 0.10 generally indicating adequate balance. Post-matching analyses accounted for the matched-pair structure. Binary outcomes were compared using McNemar’s test, while continuous outcomes were analyzed using paired tests or regression models with robust standard errors clustered by matched pair. Multivariate logistic regression analysis was performed to identify independent factors associated with live birth rate, incorporating variables with *P* < 0.100 in univariate analysis into the regression model. Odds ratios (OR) and 95% confidence intervals (CI) were calculated. No significant multicollinearity was detected among the covariates included in the final model, with all variance inflation factor (VIF) values below 5. All tests were two-sided, and *P* ≤ 0.05 was considered statistically significant.

## Results

### Baseline characteristics

**Figure 1:**
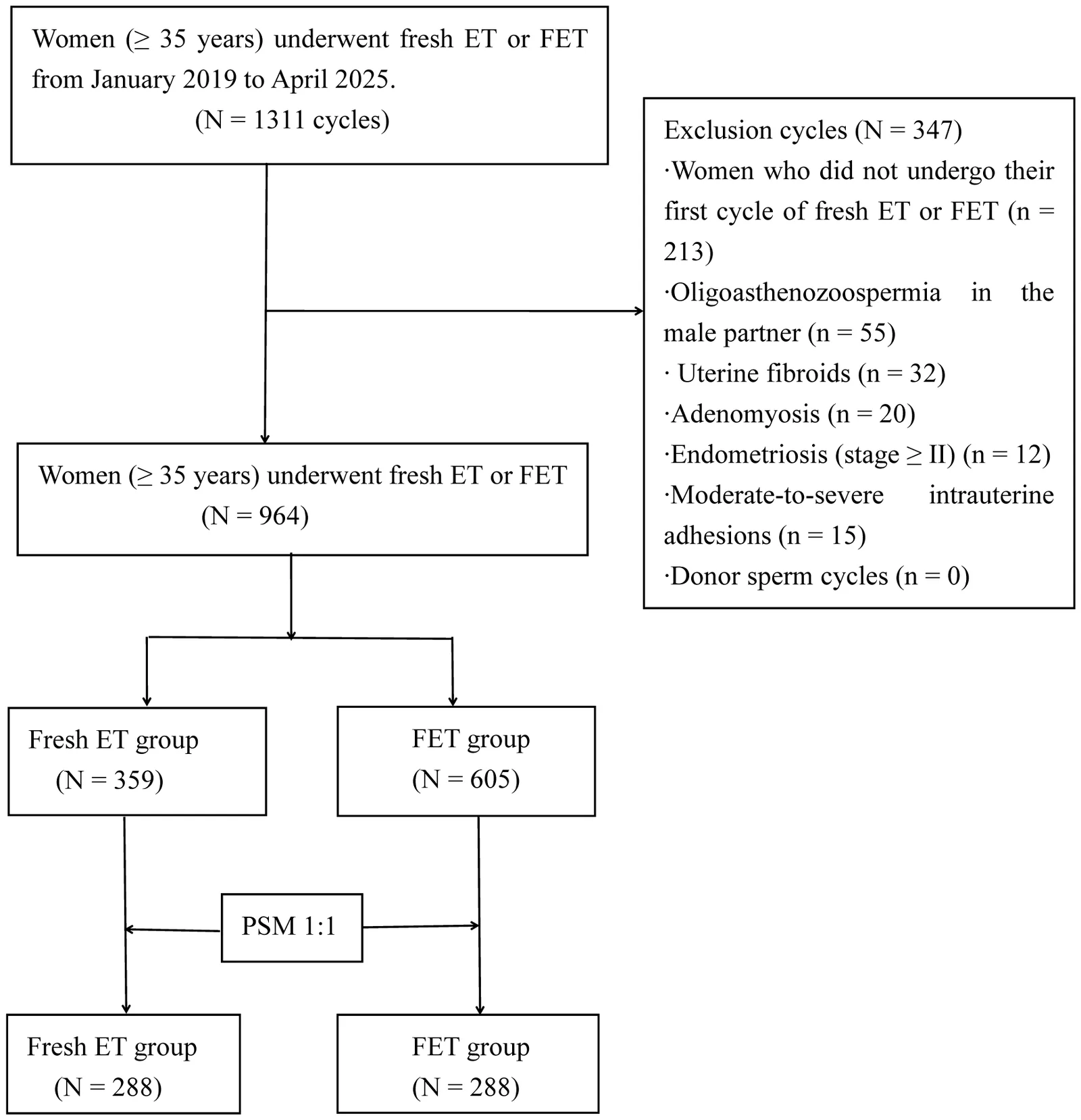
Flowchart of patient inclusion and exclusion criteria. Abbreviation: ET, embryo transfer; FET, frozen embryo transfer; PSM, propensity score matching.

A total of 1311 cycles from women of advanced age who underwent fresh ET or FET were screened. Of these, 359 fresh ET cycles and 605 FET cycles were included. Exclusion reasons were shown in **Figure 1**. After PSM at a 1:1 ratio, 288 matched cycles remained in each group. Before PSM, duration of infertility, anti-Müllerian hormone (AMH) levels, antral follicle count (AFC), endometrium thickness, endometrial morphology, blastocyst transfer rate, and number of embryos transferred were different between two groups (**Table 1**). After PSM, no significant differences were observed in any of the baseline characteristics, confirming good comparability.

**Table 1:** Baseline Characteristics between the Fresh ET and FET Groups before and after PSM.

Variables	Before PSM			After PSM		
	Fresh ET(N=359)	FET (N=605)	*P*-Value	Fresh ET (N=288)	FET (N=288)	*P*-Value
Female Age (years)	37.77±2.56	37.99±2.76	0.230	37.94±2.59	37.98±2.55	0.833
Male Age (years)	37.61±4.99	38.25±5.02	0.058	37.75±5.04	38.45±5.56	0.111
Duration of Infertility (years)	4.62±3.90	5.26±4.30	0.022	4.74±4.01	4.70±4.01	0.909
Type of Infertility [%(n/N)]			0.625			0.844
Primary	23.40 (84/359)	24.79 (150/605)		22.92 (66/288)	23.61 (68/288)	
Secondary	76.60 (275/359)	75.21 (455/605)		77.08 (222/288)	76.39 (220/288)	
Number of Previous Pregnancies	1.91±1.64	1.75±1.47	0.240	1.85±1.62	1.83±1.63	0.879
Cause of Infertility [%(n/N)]			0.217			0.411
Advanced Age and DOR	47.08 (169/359)	41.32 (250/605)		47.92 (138/288)	44.44 (128/288)	
Tubal Factor	45.50 (163/359)	50.08 (303/605)		43.40 (125/288)	48.61 (140/288)	
Ovulatory Disorder	7.52 (27/359)	8.60 (52/605)		8.68 (25/288)	6.94 (20/288)	
Treatment Year [%(n/N)]			0.055			0.075
2019	10.03 (36/359)	8.26 (50/605)		12.15 (35/288)	10.42 (30/288)	
2020	5.29 (19/359)	10.91 (66/605)		6.60 (19/288)	13.54 (39/288)	
2021	19.22 (69/359)	15.37 (93/605)		19.44 (56/288)	15.28 (44/288)	
2022	16.99 (61/359)	18.68 (113/605)		16.32 (47/288)	15.63 (45/288)	
2023	17.83 (64/359)	17.20 (104/605)		17.01 (49/288)	17.71 (51/288)	
2024	23.40 (84/359)	20.99 (127/605)		23.26 (67/288)	19.44 (56/288)	
2025	7.24 (26/359)	8.60 (52/605)		5.21 (15/288)	7.99 (23/288)	
BMI (kg/m^2^)	24.11±3.00	24.09±3.24	0.909	24.19±3.00	24.18±3.31	0.977
Basal FSH (mIU/ml)	6.76±2.56	6.64±2.95	0.494	6.66±3.01	6.91±2.76	0.288
AMH (ng/ml)	2.75±2.38	3.33±3.01	0.001	2.78±2.37	2.764±2.34	0.918
AFC	12.17±7.63	13.80±9.29	0.003	12.33±7.93	12.34±8.71	0.984
Endometrial Thickness (mm)	11.61±2.33	10.43±2.36	<0.001	11.30±2.16	11.04±2.28	0.162
Endometrial Morphology			0.023			0.186
A	0.557 (2/359)	0.992 (6/605)		0.35 (1/288)	1.39 (4/288)	
B	88.30 (317/359)	92.73 (561/605)		87.15 (251/288)	82.64 (238/288)	
C	11.14 (40/359)	6.28 (38/605)		12.50 (36/288)	15.97 (46/288)	
Blastocyst Transfer Rate	7.80 (28/359)	43.31 (262/605)	<0.001	9.38 (27/288)	7.64 (22/288)	0.455
Number of Embryos Transferred			<0.001			0.352
One	57.94 (208/359)	69.59 (421/605)		56.94 (164/288)	60.76 (175/288)	
Two	42.06 (151/359)	30.41 (184/605)		43.06 (124/288)	39.24 (113/288)	
Good-quality Embryo Transfer Cycle Rate (%)	83.84 (301/359)	84.30 (510/605)	0.852	85.07 (245/288)	87.50 (252/288)	0.397

Normally distributed continuous data were expressed as mean ± SD (Standard Deviation), and compared by independent samples t-test. Non-normally distributed continuous data were presented as median (interquartile range) [M (Q1, Q2)], and compared by Mann-Whitney U test. Categorical variables were expressed as percentage (counts) and compared using the chi-square test or Fisher’s exact test as appropriate.

PSM, propensity score matching; ET, embryo transfer; FET, frozen embryo transfer; BMI, body mass index; FSH, follicle-stimulating hormone; AMH, anti-Müllerian hormone; AFC, antral follicle count; DOR, diminished ovarian reserve.

Before matching, several covariates were imbalanced between the two groups, with absolute SMD exceeding 0.1 for male age, duration of infertility, cause of infertility, treatment year, basal FSH, AMH, AFC, endometrial thickness, embryo developmental stage, and number of embryos transferred. After PSM, all included covariates achieved good balance, with SMD values all below 0.1 (**Supplementary Table 1**).

### Pregnancy and obstetric outcomes

After PSM, the clinical pregnancy rate was higher in the fresh ET group than in the FET group (39.58% vs. 31.94%), but the difference reached borderline significance (*P* = 0.056). The live birth rate was significantly higher in the fresh ET group compared with the FET group (28.13% vs. 20.49%, *P* = 0.033). No significant differences were observed between the two groups in ectopic pregnancy rate, miscarriage rate, preterm birth rate, twin delivery rate, or cesarean section rate (all *P* > 0.05) (**Table 2**). For singleton deliveries after PSM, there were no significant differences in maternal BMI, gestational age, birth weight, newborn sex distribution, or macrosomia (all *P* > 0.05) (**Table 3**). For LBW, the number of events was extremely low (0 in the fresh ET group and 1 in the FET group); therefore, statistical comparison was not performed. For twin deliveries, the sample sizes after PSM were extremely small, with only 10 fresh ET and 4 FET cases, precluding meaningful statistical inference. The LBW rate was 75.00% (6/8) in the FET group and 30.00% (6/20) in the fresh ET group. Given the very limited number of twin deliveries, these findings should be interpreted with caution and require further validation. Other perinatal parameters, including gestational age and birth weight, showed no clear differences between the two groups (**Table 3**).

**Table 2:** Pregnancy Outcomes between the Fresh ET and FET Groups before and after PSM.

Variables	Before PSM			After PSM		
	Fresh ET (N=359)	FET (N=605)	*P*-Value	Fresh ET (N=288)	FET (N=288)	*P*-Value
Clinical Pregnancy Rate (%)	38.44 (138/359)	37.02 (224/605)	0.661	39.58 (114/288)	31.94 (92/288)	0.056
Ectopic Pregnancy Rate (%)	0.72 (1/138)	1.34 (3/224)	0.979	0.88 (1/114)	3.26 (3/92)	0.218
Miscarriage Rate (%)	27.74 (38/137)	29.86 (66/221)	0.667	28.32 (32/113)	33.71 (30/89)	0.410
Live Birth Rate (%)	27.58 (99/359)	25.62 (155/605)	0.505	28.13 (81/288)	20.49 (59/288)	0.033
Preterm Birth Rate (%)	12.12 (12/99)	12.26 (19/155)	0.974	8.64 (7/81)	10.17 (6/59)	0.758
Twin Delivery Rate (%)	13.13 (13/99)	5.81 (9/155)	0.043	12.35 (10/81)	6.78 (4/59)	0.424
Cesarean Section Rate (%)	37.37 (37/99)	25.16 (39/155)	0.038	35.8 (29/81)	22.03 (13/59)	0.079

Normally distributed continuous data were expressed as mean ± SD (Standard Deviation), and compared by independent samples t-test. Non-normally distributed continuous data were presented as median (interquartile range) [M (Q1, Q2)], and compared by Mann-Whitney U test. Categorical variables were expressed as percentage (counts) and compared using the chi-square test or Fisher’s exact test as appropriate.

PSM, propensity score matching; ET, embryo transfer; FET, frozen embryo transfer.

**Table 3:** Perinatal outcomes between the Fresh ET and FET groups before and after PSM.

Variables	Before PSM			After PSM		
	Fresh ET	FET	*P*-value	Fresh ET	FET	*P*-value
Singleton Deliveries	N = 86	N = 146		N = 71	N = 55	
Maternal BMI (kg/m^2^)	24.14 ± 3.22	23.82 ± 3.20	0.465	24.28 ± 3.25	24.18 ± 3.39	0.909
Gestational Age (weeks)	38.27 ± 1.52	37.88 ± 2.03	0.551	38.41 ± 1.15	38.38 ± 1.39	0.907
Birth Weight (g)	3410.06 ± 483.59	3383.97 ± 553.32	0.717	3440.63 ± 453.98	3475.18 ± 484.46	0.681
Newborn Sex [%(n/N)]			0.551			0.116
Female	41.86 (36/86)	45.89 (67/146)		42.25 (30/71)	56.36 (31/55)	
Male	58.14 (50/86)	54.11 (79/146)		57.75 (41/71)	43.64 (24/55)	
Macrosomia [%(n/N)]	8.14 (7/86)	11.64 (17/146)	0.397	8.45 (6/71)	14.55 (8/55)	0.280
LBW [%(n/N)]	1.16 (1/86)	4.79 (7/146)	0.143	0 (0/71)	1.8 (1/55)	–
Twin Deliveries	N = 13	N = 9		N = 10	N = 4	
Maternal BMI (kg/m^2^)	23.135 ± 2.25	25.02 ± 2.39	0.074	23.67 ± 1.86	24.53 ± 3.58	–
Gestational Age (weeks)	35.31 ± 3.88	35.33 ± 1.66	0.958	35.30 ± 4.11	35.75 ± 0.96	–
Birth weight (g)	2441.35 ± 672.97	2533.89 ± 390.22	0.301	2479.25 ± 704.00	2363.75 ± 331.62	–
Newborn Sex [%(n/N)]			0.325			–
Female	57.69 (15/26)	72.22 (13/18)		60.00 (12/20)	62.50 (5/8)	
Male	42.31 (11/26)	27.78 (5/18)		40.00 (8/20)	37.50 (3/8)	
Macrosomia [%(n/N)]	0	0	–	0	0	–
LBW [%(n/N)]	42.31 (11/26)	61.11 (11/18)	--	30.00 (6/20)	75.00(6/8)	–

Normally distributed continuous data were expressed as mean ± SD (Standard Deviation), and compared by independent samples t-test. Non-normally distributed continuous data were presented as median (interquartile range) [M (Q1, Q2)], and compared by Mann-Whitney U test. Categorical variables were expressed as percentage (counts) and compared using the chi-square test or Fisher’s exact test as appropriate.

PSM, propensity score matching; ET, embryo transfer; FET, frozen embryo transfer; LBW, low birth weight.

For twin deliveries in the “After PSM” cohort, the sample sizes were too small (fresh ET: N = 10; FET: N = 4) to perform meaningful statistical tests; therefore, *P*-values were not provided and data are presented descriptively only.

### Univariate analysis and multivariate regression analysis

Given that the live birth rate differed significantly between the two groups after PSM (**Table 2**), we further performed univariate and multivariate analyses to identify factors associated with live birth rate. As shown in **Table 4**, univariate comparisons between the live birth group and the no live birth group revealed that female age, male age, infertility factors, AFC, number of embryos transferred, and transfer strategy were significantly associated with live birth (all *P* < 0.05). AMH showed a trend towards association with live birth (*P* = 0.051), and good-quality embryo transfer cycle rate reached borderline significance (*P* = 0.080). Variables with *P* < 0.100 in univariate analysis were entered into the multivariate model (**Table 5**). Multicollinearity was assessed using the variance inflation factor, and no significant multicollinearity was detected among the covariates included in the final model. Model calibration was acceptable according to the Hosmer–Lemeshow goodness-of-fit test (χ^2^ = 13.530, df = 8, *P* = 0.095). After adjustment, female age remained a significant negative predictor of live birth (*P* < 0.001). Transfer of two embryos and good-quality embryo transfer cycle rate were associated with higher live birth rate (all *P* < 0.05). Importantly, after adjusting, the transfer strategy (fresh ET or FET) did not reach statistical significance, although it showed a borderline trend (*P* = 0.072). Male age, infertility factors, AMH, AFC, endometrial thickness, and blastocyst transfer rate were not predictors independently associated with live birth (all *P* > 0.05).

**Table 4:** Univariate analysis of live birth after PSM.

Variables	Univariate analysis		
	Live birth group (N = 140)	No live birth group (N = 436)	*P*-value
Female Age (years)	36.94 ± 1.81	38.29 ± 2.69	< 0.001
Male Age (years)	36.59 ± 5.29	38.59 ± 5.23	< 0.001
Years of Infertility (years)	5.15 ± 4.18	4.58 ± 3.94	0.142
Type of Infertility [%(n/N)]			0.557
Primary	24.29 (34/140)	22.02 (96/436)	
Secondary	75.71 (106/140)	77.98 (340/436)	
Cause of Infertility [%(n/N)]			0.019
Advanced Age and DOR	36.43 (51/140)	49.08 (214/436)	
Tubal Factor	57.14 (80/140)	43.58 (190/436)	
Ovulatory Disorder	6.43 (9/140)	7.34 (32/436)	
Year of Treatment			0.428
2019	11.43 (16/140)	11.47 (50/436)	
2020	7.14 (10/140)	13.07 (57/436)	
2021	22.86 (32/140)	16.28 (71/436)	
2022	14.29 (20/140)	15.83 (69/436)	
2023	17.86 (25/140)	17.20 (75/436)	
2024	19.29 (27/140)	19.04 (83/436)	
2025	7.14 (10/140)	7.11 (31/436)	
BMI (kg/m2)	24.204 ± 3.211	24.180 ± 3.238	0.939
Basal FSH (mIU/ml)	6.473 ± 2.568	6.883 ± 2.978	0.143
AMH (ng/ml)	3.111 ± 2.551	2.666 ± 2.276	0.051
AFC	13.59 ± 7.852	11.93 ± 8.436	0.040
Endometrial Thickness	11.486 ± 2.295	11.073 ± 2.192	0.056
Endometrial Morphology			0.830
A	0.71 (1/140)	0.92 (4/436)	
B	83.57 (117/140)	85.32 (372/436)	
C	15.71 (22/140)	13.76 (60/436)	
Blastocyst Transfer Rate	10.00 (14/140)	8.03 (35/436)	0.467
Number of Embryos Transferred			<0.001
One	45.00 (63/140)	63.30 (276/436)	
Two	55.00 (77/140)	36.70 (160/436)	
Good-quality Embryo Transfer Cycle Rate	90.71 (127/140)	84.86 (370/436)	0.08
Transfer Strategy			0.033
Fresh ET	57.86 (81/140)	47.48 (207/436)	
FET	42.14 (59/140)	52.52 (229/436)	

Normally distributed continuous data were expressed as mean ± SD (Standard Deviation), and compared by independent samples t-test. Non-normally distributed continuous data were presented as median (interquartile range) [M (Q1, Q2)], and compared by Mann-Whitney U test. Categorical variables were expressed as percentage (counts) and compared using the chi-square test or Fisher’s exact test as appropriate.

PSM, propensity score matching; ET, embryo transfer; FET, frozen embryo transfer; BMI, body mass index; FSH, follicle-stimulating hormone; AMH, anti-Müllerian hormone; AFC, antral follicle count; DOR, diminished ovarian reserve.

**Table 5:** Multivariate logistic regression analysis of live birth after PSM.

Variables	Multivariate analysis		
	B value	OR(95%CI)	*P*-value
Female Age (years)	- 0.23	0.80 (0.71 – 0.89)	< 0.001
Male Age (years)	- 0.02	0.98 (0.93 – 1.03)	0.372
Cause of Infertility			0.322
Tubal Factor vs. Advanced Age and DOR	0.03	1.03 (0.64 – 1.67)	
Ovulatory Disorder vs. Advanced Age and DOR	- 0.62	0.54 (0.21 – 1.36)	
AMH (ng/ml)	0.05	1.05 (0.94 – 1.19)	0.384
AFC	- 0.001	1.00 (0.97 – 1.03)	0.965
Endometrial Thickness (mm)	0.07	1.07 (0.98 – 1.17)	0.158
Number of Embryos Transferred (2 vs. 1)	0.86	2.35 (1.53 – 3.63)	<0.001
Good-quality Embryo Transfer Cycle Rate	0.81	2.25 (1.15–4.41)	0.017
Fresh ET vs. FET	0.38	1.46 (0.97 – 2.21)	0.072

PSM, propensity score matching; OR, odds ratios; CI, confidence intervals; ET, embryo transfer; FET, frozen embryo transfer; AMH, anti-Müllerian hormone; AFC, antral follicle count; DOR, diminished ovarian reserve.

### Conditional logistic regression

To further account for the paired nature of the data after propensity score matching, we performed a sensitivity analysis using conditional logistic regression stratified by matched pair ID. A total of 108 discordant matched pairs were identified and included in this analysis. The results were partially consistent with the primary multivariable analysis. Female age remained a significant negative predictor of live birth (*P* = 0.034), and transfer of two embryos remained a strong positive predictor (*P* = 0.005). However, good-quality embryo transfer cycle rate was no longer significantly associated with live birth in this sensitivity analysis (*P* = 0.956), which may be attributable to the reduced sample size, as only discordant pairs contributed to the conditional estimates. Importantly, the transfer strategy (fresh ET vs. FET) remained non-significant (*P* = 0.344), confirming the robustness of our primary finding that the choice between fresh and frozen embryo transfer is not an independent predictor of live birth in women of advanced age (**Supplementary Table 2**).

## Discussion

The optimal transfer strategy in women of advanced age remains a subject of ongoing debate. In this PSM study comparing fresh ET with FET in women of advanced age, fresh ET tended to have a higher clinical pregnancy rate and live birth rate than FET, though the difference was not statistically significant. Chen et al. reviewed 720 women of advanced age undergoing their first fresh ET or FET and found no significant difference in live birth rate between the two groups, although the clinical pregnancy rate was significantly higher in the fresh ET group (10). They concluded that FET may not be beneficial for women of advanced age. Similarly, Sun et al. conducted a PSM study including women aged 35–45 years undergoing their first fresh or frozen cleavage-stage embryo transfer (11). They concluded that FET did not improve live birth rate and other clinical outcomes as compared with fresh ET. Lattes et al. conducted a retrospective cohort study of 1882 first embryo transfer cycles stratified by age. They found that live birth rates were significantly higher for FET in women under 35 years, but not in women over 35 years (12). A large UK multicenter RCT, not stratified by age, found no significant differences in clinical pregnancy, live birth, or miscarriage rates between the fresh ET and FET (13). However, a retrospective cohort study showed that live birth rate was greater for first FET than fresh ET with advancing age (14). They thought the presence of a more favorable age-related change in endometrial receptivity present in frozen-thawed cycles. Our study extended these findings by demonstrating that although fresh ET showed a significantly higher live birth rate in univariate analysis after PSM, this advantage was largely explained by younger female age, higher number of embryos transferred, and higher proportion of good-quality embryos, rather than by transfer strategy itself.

Our finding that female age was the strongest negative predictor of live birth rate. Female fertility declines significantly after age 35 and more rapidly after age 40, primarily due to age-related decline in ovarian reserve and oocyte competence (15). A study of 4958 infertile women using a freeze-all strategy demonstrated that live birth rate for the first FET cycle significantly declined with increasing female age, particularly among those aged 35–37 and 38–40 years (16). The dramatic decline across age groups underscores the overriding influence of maternal age on reproductive outcomes. Our study fully corroborates this finding, and this influence consistently outweighs the impact of transfer strategy choice.

Our finding that transfer of two embryos versus one was the positive predictor of live birth rate. We also found that there was no significant difference in twin delivery rates between the two transfer strategies. However, the benefit of double-embryo transfer must be weighed against the increased risks of multiple gestation, which are well documented to increase maternal and neonatal morbidity (17). Although transfer of two embryos was associated with higher odds of live birth in the present analysis, this finding should not be interpreted as a recommendation for routine double-embryo transfer. The number of embryos transferred should be individualized according to maternal age, embryo stage and quality, reproductive prognosis, and the risks associated with multiple gestation (18).

For singleton deliveries, we observed no significant differences between fresh ET and FET in gestational age, birth weight, or other perinatal parameters. Chen et al. found that FET resulted in higher birthweights compared with fresh ET in women of advanced age (10). Our study did not detect this difference, possibly because of the relatively small sample size in the singleton deliveries. For twin deliveries, however, the FET group had a significantly higher low birth weight rate than the fresh ET group. This finding was based on an extremely small sample, which renders the statistical robustness of this result questionable.

### Study limitations

The incidence of other pregnancy-related complications, such as gestational diabetes, hypertensive disorders, anemia, hypothyroidism, oligohydramnios, premature rupture of membranes, and postpartum hemorrhage, was too low in our cohort to permit meaningful statistical analysis. This may be partly attributable to incomplete or insufficiently detailed follow-up data, highlighting the need for more rigorous and comprehensive documentation of maternal and neonatal complications in future studies to thoroughly evaluate the safety profiles of the two strategies. Despite the use of PSM to control for measurable confounders, inherent selection bias of a retrospective design may persist, including unmeasured confounders such as clinician preference and patient choice. The relatively small sample size, particularly in the twin delivery subgroup, limits the reliability of the perinatal conclusions. In addition, the single-center nature of the study may limit the generalizability of our findings.

## Conclusions

In women of advanced age, live birth rates were comparable between FET and fresh ET after adjustment for confounders, and transfer strategy was not an independent predictor of live birth. Female age, number of embryos transferred, and good-quality embryo transfer cycle rate were the important determinants of live birth. Notably, the freeze-all strategy did not appear to confer additional benefit over fresh ET in women of advanced age. Therefore, clinical decision-making should prioritize patient age assessment, embryo quality optimization, and individualized determination of the number of embryos to transfer, rather than focusing predominantly on the choice between fresh ET and FET. Future prospective studies with larger sample sizes and more detailed follow-up protocols are warranted to further validate these findings and to comprehensively assess perinatal and maternal outcomes, particularly in twin deliveries.

## Data Availability

The original contributions presented in the study are included in the article/**Supplementary Material**, further inquiries can be directed to the corresponding authors.
