# Supplementary material for: Pregnancy outcomes after fresh and frozen embryo transfer in women of advanced age and analysis of influencing factors: single-center experience
Source: Front Endocrinol (Lausanne). 2026 Aug 12;17:1883081. doi: 10.3389/fendo.2026.1883081 (PMC13506357; doi:10.3389/fendo.2026.1883081)
Supplement: Supplementary file 1 [file Supplementaryfile1.docx]

Supplementary Table S1 Covariate Balance before and after PSM

| Covariate | Absolute SMD before PSM | Absolute SMD after PSM |
| --- | --- | --- |
| Female Age | 0.08 | 0.02 |
| Male Age | 0.12 | 0.01 |
| Duration of Infertility | 0.16 | 0.01 |
| Type of Infertility | 0.04 | 0.02 |
| Cause of Infertility | 0.11 | 0.05 |
| Treatment Year | 0.25 | 0.01 |
| BMI | 0.01 | 0.04 |
| Basal FSH | 0.16 | 0.07 |
| AMH | 0.25 | 0.06 |
| AFC | 0.21 | 0.04 |
| Endometrial Thickness | 0.51 | 0.06 |
| Embryo Developmental Stage | 1.32 | 0.04 |
| Number of Embryos Transferred | 0.24 | 0.08 |
| Embryo Morphological Grade | 0.02 | 0.02 |

Absolute standardized mean differences (SMDs) were used to assess covariate balance before and after propensity score matching. An absolute SMD < 0.10 was considered indicative balance. All prespecified covariates achieved adequate balance after matching.

Abbreviations: PSM, propensity score matching; BMI, body mass index; FSH, follicle-stimulating hormone; AMH, anti‑Müllerian hormone; AFC, antral follicle count.

Supplementary Table S2 Conditional Logistic Regression for Factors Associated with Live Birth after PSM

| Variable | OR (95%CI) | *P*-Value |
| --- | --- | --- |
| Female Age | 0.82（0.68-0.99） | 0.034 |
| Male Age | 0.95（0.87-1.02） | 0.174 |
| AMH | 1.11（0.90-1.36） | 0.327 |
| AFC | 1.00（0.95-1.05） | 0.941 |
| Endometrial Thickness | 1.15（0.88-1.50） | 0.309 |
| Number of Embryos transferred (2 vs. 1) | 2.90（1.39-6.06） | 0.005 |
| Good-quality Embryo Transfer Cycle Rate | 0.97（0.33-2.85） | 0.956 |
| Transfer Strategy (Fresh ET vs. FET) | 1.23（0.80-1.91） | 0.344 |

Conditional logistic regression was performed on 108 discordant matched pairs identified after PSM. The analysis was stratified by matched pair ID to account for the paired study design. Variables included in the model were those showing clinical relevance or statistical significance in multivariate analysis. Model discrimination was assessed by the concordance statistic (C-index).

Abbreviations: PSM, propensity score matching; OR, odds ratios; CI, confidence intervals; ET, embryo transfer; FET, frozen embryo transfer; AMH, anti‑Müllerian hormone; AFC, antral follicle count.
